# Statistical optimization of a novel excipient (CMEC) based gastro retentive floating tablets of propranolol HCl and it’s *in vivo* buoyancy characterization in healthy human volunteers

**DOI:** 10.1186/2008-2231-20-21

**Published:** 2012-08-30

**Authors:** Venkata Srikanth Meka, Sreenivasa Rao Nali, Ambedkar Sunil Songa, Janaki Ram Battu, Venkata Ramana Murthy Kolapalli

**Affiliations:** 1 School of Pharmacy, International Medical University, Kuala Lumpur, 57000, Malaysia; 2 A.U. College of Pharmaceutical Sciences, Andhra University, Visakhapatnam, 530003, India

**Keywords:** Propranolol HCl, Gastro retentive, Floating, Central composite, Carboxymethyl ethyl cellulose

## Abstract

The objective of the present investigation is to formulate gastro retentive floating drug delivery systems (GRFDDS) of propranolol HCl by central composite design and to study the effect of formulation variables on floating lag time, D_1hr_ (% drug release at 1 hr) and t_90_ (time required to release 90% of the drug). 3 factor central composite design was employed for the development of GRFDDS containing novel semi synthetic polymer carboxymethyl ethyl cellulose (CMEC) as a release retarding polymer. CMEC, sodium bicarbonate and Povidone concentrations were included as independent variables. The tablets were prepared by direct compression method and were evaluated for *in vitro* buoyancy and dissolution studies. From the polynomial model fitting statistical analysis, it was confirmed that the response floating lag time and D_1hr_ is suggested to quadratic model and t_90_ is suggested to linear model. All the statistical formulations followed first order rate kinetics with non-Fickian diffusion mechanism. The desirability function was used to optimize the response variables, each having a different target, and the observed responses were highly agreed with experimental values. Statistically optimized formulation was characterized by FTIR and DSC studies and found no interactions between drug and polymer. The results demonstrate the feasibility of the model in the development of GRFDDS containing a propranolol HCl. Statistically optimized formulation was evaluated for *in vivo* buoyancy studies in healthy humans for both fed and fasted states. From the results, it was concluded that gastric residence time of the floating tablets were enhanced at fed stage but not in fasted state.

## Introduction

Drug delivery systems (DDS) are used for maximizing the therapeutic index of the drug and also targeted for reduction in the side effects. All over delivery systems the oral drug delivery has become the mainstay of treatment due to higher patient compliance and reduced patient discomfort. Under certain circumstances prolonging the gastric retention of a DDS is desirable for achieving greater therapeutic benefit of the drug [1]. For example, drugs that are absorbed in the proximal part of the gastrointestinal tract (GIT), and the drugs that are less soluble or are degraded by the alkaline pH may benefit from prolong gastric retention. In addition, for local and sustained drug delivery to the stomach and the proximal small intestine to treat certain conditions, prolonging gastric retention of the therapeutic moiety may offer numerous advantages including improved bioavailability, therapeutic efficacy and possible reduction of the dose size [2-4]. All over the retentive systems gastric floating system for modulation of oral controlled drug delivery was found to be great importance. Hence in the present investigation effervescent floating systems were developed for prolonging the gastric retention.

In the present investigation propranolol HCl was selected as a model drug for the development of gastro retentive floating drug delivery systems (GRFDDS). Propranolol is a nonselective beta-adrenergic receptor blocking agent possessing no other autonomic nervous system activity used for the treatment of hypertension [5]. It is highly lipophilic and almost completely absorbed after oral administration. However, it undergoes high first-pass metabolism by the liver and on average, only about 25% of propranolol reaches the systemic circulation [6]. Variability of propranolol bioavailability is depends upon the secretory transporter P-glycoprotein (P-gp) located on the epithelium cells. Although P-gp appears to be distributed throughout the GIT, its levels are higher in more distal regions (stomach < jejunum < colon). Absorption through P-gp prolongs the drug exposure to CYP3A4. The colocalization of P-gp and CYP3A4 in the mature enterocytes and their overlapping substrate specificity reasonably suggests that the function of these two proteins may be synergistic and appear to be coordinately regulated. Consequently, a greater proportion of drug will be metabolized since the repetitive two-way kinetics (drug excerption from the enterocytes into the lumen via P-gp and reabsorption back into enterocytes) will simply prolong the drug exposure to CYP3A4. This mechanism not only limits the absorption of a wide variety of drugs, including peptides, but also poses a threat for potential drug interactions [7,8].

Based on previously published literature, applications of gastro retentive drug delivery system (GRDDS) may be suitable for the drugs insoluble in intestinal fluids (acid soluble basic drugs), e.g., propranolol, metoprolol, diazepam [8]. As discussed earlier, propranolol has short half-life, high first-pass metabolism, presence of food increases the bioavailability, P-gp plays important role in the absorption, and the drug is acid-soluble basic drug which make it suitable for GRDDS. A novel semi synthetic polymer carboxy methyl ethyl cellulose (CMEC) was used as release retarding polymer in the present investigation. Till now there were no reports found on CMEC as a release retarding polymer.

In the normal conventional optimization process, a single independent variable is varied while all others are kept constant at a specific set of conditions. It’s not possible to change more than one parameter at a time during the formulation development. This method may lead to unreliable results and improper conclusions besides wastage of excipients due to the requirement of large number of runs in achieving the desired goal. Response surface methodology (RSM) is an alternative to overcome this difficulty, which can be employed to optimize the formulations with suitable experimental design. RSM permits a deeper understanding of a process or product and has important applications like optimization and in establishing the robustness of that product. Central composite designs are a progression from the factorial designs which have been widely used in response-surface modeling and optimization [9].

The objective of the present investigation is to develop gastro retentive floating tablets (GRFT) of propranolol HCl using central composite design. In this study CMEC quantity, sodium bicarbonate concentration and Povidone concentration were selected as independent variables while floating lag time, D_1hr_ and t_90_ were selected as dependent variables. For this study Design Expert software was used which gives information regarding critical values for achieving the desired response and also the possible interaction effects of selected independent variables on dependent variable.

## Experimental

### Materials

Propranolol HCl was provided by Dr Reddy’s Laboratories Ltd (Hyderabad, India). CMEC, sodium bicarbonate, Povidone K 30 and magnesium stearate were obtained as gift samples from Unichem Laboratories Ltd (Goa, India). All other reagents and chemicals were of analytical grade.

### Experimental design

RSM is an experimental design technique by which the factors involved and their relative importance can be assessed. In the present study, a central composite design was employed containing 3 factors evaluated at 3 levels and experimental trials were performed at all 20 possible combinations. The levels of the 3 independent variables are shown in Table 1 and the formulation variables evaluated include:

**Table 1:** Experimental range and levels of the independent variables in CMEC based formulations

**Variables**	**Range and levels**		
	**-1**	**0**	**+1**
CMEC (mg) X_1_	200	240	280
% w/w Sodium bicarbonate concentration *X*_2_	5	10	15
% w/w povidone concentration X_3_	2.5	5	7.5

X_1_ = CMEC quantity in mg

*X*_2_ = % w/w Sodium bicarbonate concentration (% w/w to the tablet weight)

X_3_ = % w/w Povidone concentration (% w/w to the tablet weight)

The response variables include

Y_1_ = Floating lag time (sec)

Y_2_ = D_1hr_ (% drug released at 1 hr)

Y_3_ = t_90_ (time required to release 90% of the drug)

#### Preparation of GRFT of propranolol HCl

All the ingredients sufficient for a batch of 100 tablets according to the formulae suggested by Design Expert software shown in Table 2 were accurately weighed and passed through the sieve 40. Propranolol HCl (80 mg) was geometrically mixed with CMEC until a homogeneous blend was achieved. Povidone and sodium bicarbonate was added to the above mixture and mixed for 5 min in a polybag. Blend was lubricated with presifted magnesium stearate (sieve 60) for 3 min in polybag. 1%w/w of magnesium stearate was used in all the formulations. The flow property of the final blend was found to be good so final blend was directly compressed into tablets on a 16-station rotary tablet punching machine (M/s. Cad mach Machinery Co. Pvt., Ltd., India) using 9 mm round plain punches.

**Table 2:** Formulations with the levels of independent variables and observed responses

**Standard Order**	**CMEC quantity (mg) X**_**1**_	**%w/w of Sodium bicarbonate** ***X***_**2**_	**%w/w of povidone X**_**3**_	**Observed responses**		
				**Floating lag time (sec)**	**D**_**1hr**_**(%)**	**t**_**90**_**(hr)**
PCMECR 01	200	5	2.5	650	40.22	6.6
PCMECR 02	280	5	2.5	550	31.11	8
PCMECR 03	200	15	2.5	720	36.58	8.2
PCMECR 04	280	15	2.5	510	18.99	10.8
PCMECR 05	200	5	7.5	300	32.12	8
PCMECR 06	280	5	7.5	600	23.45	9.9
PCMECR 07	200	15	7.5	495	22.12	9.9
PCMECR 08	280	15	7.5	670	13.84	12.2
PCMECR 09	172.73	10	5	570	27.23	9
PCMECR 10	307.27	10	5	369	18.12	11
PCMECR 11	240	1.59	5	450	28.15	8.85
PCMECR 12	240	18.41	5	300	18.99	12
PCMECR 13	240	10	0.80	310	34.59	9.8
PCMECR 14	240	10	9.20	280	26.68	11.15
PCMECR 15	240	10	5	312	19.12	10.3
PCMECR 16	240	10	5	369	18.99	10.4
PCMECR 17	240	10	5	435	19.89	10.25
PCMECR 18	240	10	5	401	19.99	10.2
PCMECR 19	240	10	5	467	19.01	10.15
PCMECR 20	240	10	5	420	18.78	10

### Evaluation of GRFT

#### *In vitro* buoyancy studies

All the formulated floating tablets (n = 5) were subjected to *in vitro* buoyancy studies. The floating lag time was determined in one liter glass beaker containing 900 ml of 0.1 N HCl [10]. The time required for the tablet to rise to the surface and float was determined as floating lag time. Results are given in Table 2.

#### *In vitro* dissolution studies

**Figure 1:**
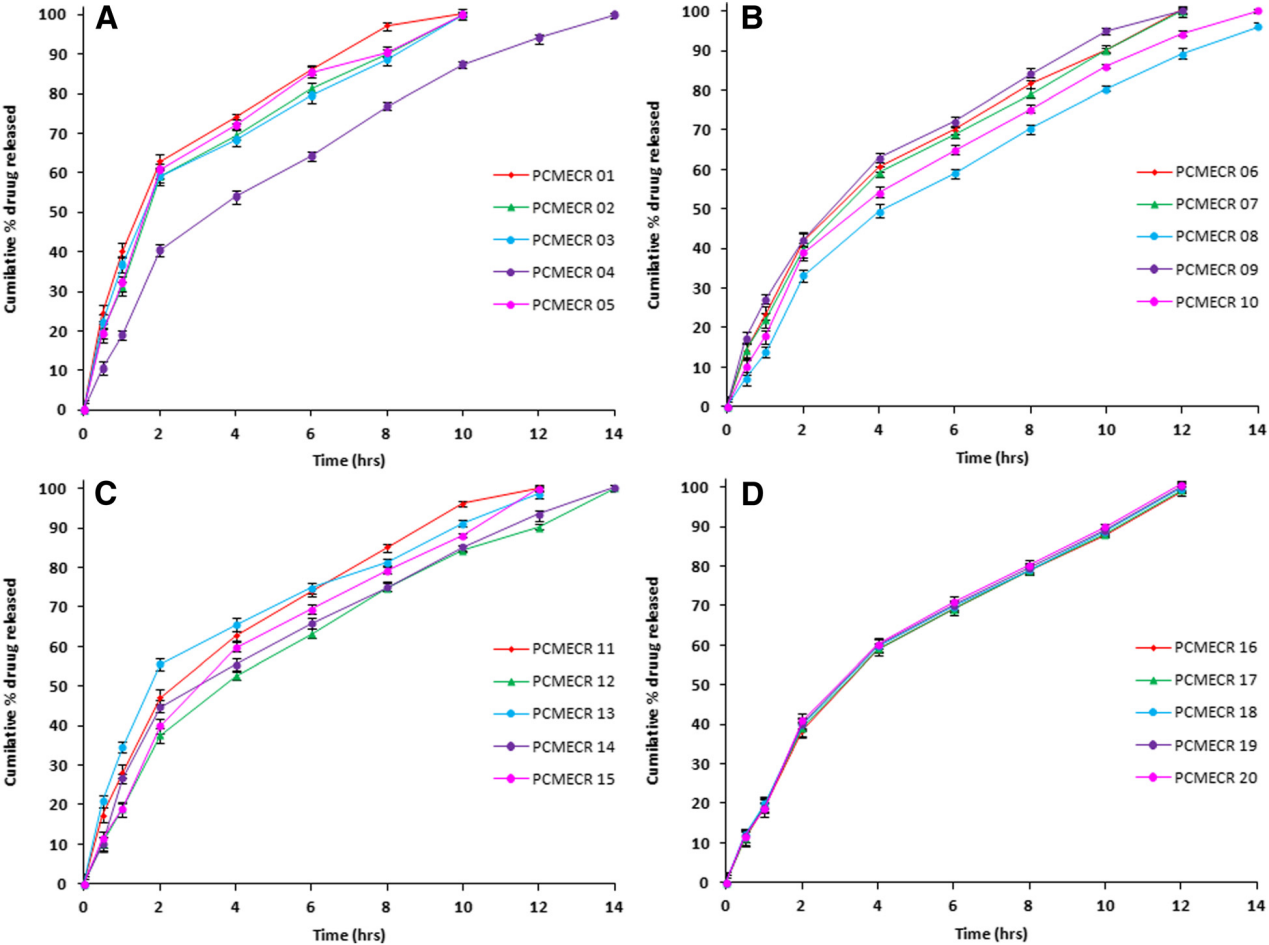
Dissolution Profile of GRFT formulations: A) PCMECR 01-05, B) PCMECR 06-10, C) PCMECR 11-15, D) PCMECR 16-20.

*In vitro* release of propranolol hydrochloride from the prepared floating tablets was studied using USP XXIII dissolution test apparatus (LABINDIA, Disso 2000) employing the paddle stirrer (Apparatus-II). 900 ml of 0.1 N HCl was used as dissolution medium maintained at a temperature of 37 ± 0.5 °C and the paddle was rotated at 50 rpm [11]. Aliquots (5 ml each) were withdrawn at predetermined time intervals by means of a syringe fitted with 0.45 μm prefilter and immediately replaced with 5 ml of fresh medium maintained at 37 ± 0.5 °C. The filtered samples were suitably diluted with the dissolution medium wherever necessary and the absorbance of the samples was measured at 289 nm and results are given in Figure 1.

### Release kinetics

#### Zero order release kinetics

Drug dissolution from dosage forms that do not disaggregate and release the drug slowly can be represented by the equation:

(1) $${\text{Q}}_{\text{t}}={\text{Q}}_{0}+{\text{K}}_{0}\text{t}$$

Where Q_t_ is the amount of drug dissolved in time *t,* Q_0_ is the initial amount of drug in the solution (most times, Q_0_ *=* 0) and K_0_ is the zero order release constant expressed in units of concentration/time [12].

#### First order release kinetics

The release of the drug which followed first order kinetics can be expressed by the equation [13]:

(2) $$\frac{dC}{dt}=-Kc$$

Where *K* is first order rate constant expressed in units of time^-1^.

This equation can be modified as

(3) $$\text{log C}=\text{log}\;{\text{C}}_{0}-\text{Kt}/2.303$$

Where C_0_ is the initial concentration of drug, k is the first order rate constant, and t is the time. The data obtained are plotted as log cumulative percentage of drug remaining vs. time which would yield a straight line with a slope of -K/2.303.

#### Higuchi equation

It defines a linear dependence of the active fraction released per unit of surface (Q) on the square root of time.

(4) $$\text{Q}=k_{2}t^{1/2}$$

Where, k_2_ is the release rate constant.

A plot of the fraction of drug released against square root of time will be linear if the release obeys Higuchi equation. This equation describes drug release as a diffusion process based on the Fick’s law, square root time dependent [14].

#### Korsmeyer-Peppas model

In order to define a model, which would represent a better fit for the formulation, dissolution data was further analyzed by Peppas and Korsmeyer equation (Power law).

(5) $$M_{t}/M_{\propto }=k.t^{n}$$

Where, M_t_ is the amount of drug released at time t and M_∝_ is the amount released at time∝, thus the M_t_/M_∝_ is the fraction of drug released at time t, k is the kinetic constant and n is the diffusion exponent.

In this model, the value of *n* characterizes the release mechanism of drug. For the case of cylindrical tablets, 0.45 = n corresponds to a Fickian diffusion mechanism, 0.45 < n < 0.89 to non-Fickian transport, n = 0.89 to Case II (relaxation) transport, and n > 0.89 to super case II transport [15].

#### Hixson - Crowell model

Hixson and Crowell recognized that the particles regular area is proportional to the cube root of its volume. They derived the equation:

(6) $$\left(W_{0}^{1/3}-W_{t}^{1/3}\right)=Kt$$

Where W_0_ is the initial amount of drug in the pharmaceutical dosage form, W_t_ is the remaining amount of drug in the pharmaceutical dosage form at time t and K (kappa) is a constant incorporating the surface – volume relation [16]. The equation describes the release from systems where there is a change in surface area and diameter of particles or tablets.

Correlation coefficients and release rate kinetics are shown in the Table 3.

**Table 3:** Correlation coefficient values and release kinetics of GRFT

**Formulation**	**Zero order**		**First order**		**Higuchi**	**Hixson Crowell**	**Peppas**	
	**Ko**	**r**	**K**_**1**_	**r**	**r**	**r**	**n**	**r**
PCMECR 01	8.8464	0.9128	0.3922	0.9764	0.9856	0.9848	0.3821	0.9837
PCMECR 02	8.9460	0.9311	0.2738	0.9912	0.9889	0.9786	0.4640	0.9687
PCMECR 03	8.6046	0.9276	0.2519	0.9872	0.9893	0.9593	0.4007	0.9822
PCMECR 04	6.8619	0.9641	0.2158	0.9860	0.9955	0.9953	0.5905	0.9841
PCMECR 05	9.0042	0.9211	0.2886	0.9908	0.9835	0.9745	0.4530	0.9644
PCMECR 06	7.7720	0.9623	0.2174	0.9955	0.9969	0.9937	0.5565	0.9910
PCMECR 07	7.7709	0.9676	0.2119	0.9916	0.9972	0.9938	0.5791	0.9920
PCMECR 08	6.1913	0.9695	0.2027	0.9728	0.9950	0.9947	0.6678	0.9827
PCMECR 09	7.7972	0.9597	0.2660	0.9778	0.9981	0.9930	0.5213	0.9966
PCMECR 10	6.8746	0.9658	0.2119	0.9831	0.9957	0.9947	0.6067	0.9844
PCMECR 11	7.7522	0.9528	0.2840	0.9709	0.9968	0.9908	0.4961	0.9926
PCMECR 12	6.7415	0.9679	0.1835	0.9959	0.9965	0.9958	0.5917	0.9896
PCMECR 13	7.0102	0.9243	0.2830	0.9463	0.9881	0.9807	0.3870	0.9845
PCMECR 14	6.5653	0.9549	0.2004	0.9822	0.9942	0.9893	0.4732	0.9927
PCMECR 15	7.8594	0.9646	0.2036	0.9965	0.9943	0.9564	0.6207	0.9826
PCMECR 16	7.8342	0.9659	0.2929	0.9975	0.9942	0.9837	0.6264	0.9854
PCMECR 17	7.8163	0.9655	0.2022	0.9967	0.9950	0.9801	0.6101	0.9869
PCMECR 18	7.8216	0.9644	0.2063	0.9957	0.9951	0.9749	0.6073	0.9852
PCMECR 19	7.8945	0.9643	0.2096	0.9958	0.9944	0.9931	0.6246	0.9820
PCMECR 20	7.9658	0.9637	0.2169	0.9950	0.9941	0.9934	0.6288	0.9798

### Statistical analysis of the data and optimization

Polynomial models including linear, interaction and quadratic terms were generated for all the response variables using Design Expert software. The best fitting model was selected based on the comparisons of several statically parameters including the coefficient of variation (CV), the coefficient of determination (R^2^), adjusted coefficient of determination (adjusted R^2^) and the predicted residual sum of square (PRESS) provided by Design Expert software. In addition, statistical analysis like analysis of variance (ANOVA) to identify significant effect of factors on response, regression coefficients, F test and P value were also calculated with the software. The results are given in Table 4-5.

**Table 4:** Summary of ANOVA results in analyzing lack of fit (LOF) and pure error

**Parameters**	**Sum of squares**	**df**	**Mean Square**	**F value**	**p value Prob > F**	**Remark**
**Floating lag time (Quadratic model)**						
Model	171694	9	19077.11	1.122	0.4269	Not significant
Residual	170028	10	17002.78			
Lack of Fit	155211	5	31042.10	10.47	0.0111	significant
Pure Error	14817	5	2963.4667			
**D**_**1hr**_**(Quadratic model)**						
Model	942.28	9	104.6973	16.04	< 0.0001	significant
Residual	65.26	10	6.5265			
Lack of Fit	63.96	5	12.7915	48.93	0.0003	significant
Pure Error	1.31	5	0.2614			
**t**_**90**_**(Linear model)**						
Model	29.439	3	9.812	21.28	< 0.0001	significant
Residual	7.377	16	0.461			
Lack of Fit	7.2837	11	0.662	35.47	0.0005	significant
Pure Error	0.0933	5.0000	0.018			

**Table 5:** Statistical parameters

**Parameters**	**Floating lag time**	**D**_**1hr**_	**t**_**90**_
Std. Dev.	130.39	2.555	0.68
Mean	458.90	24.399	9.84
C.V. %	28.41	10.471	6.90
PRESS	1195095.90	502.960	12.95
R-Squared	0.5024	0.9352	0.7996
Adj R-Squared	0.0546	0.8769	0.7621
Pred R-Squared	-2.4973	0.5008	0.6484
Adeq Precision	3.4934	12.842	16.4605

**Figure 2:**
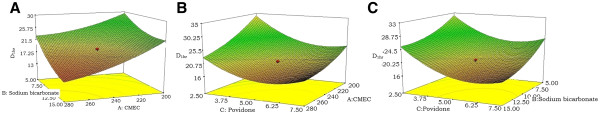
**Response surface plot for (A) the effect of CMEC and sodium bicarbonate concentrations on D**_**1hr**_**(B) effect of CMEC and povidone concentrations on D**_**1hr**_**(C) effect of sodium bicarbonate and povidone concentrations on D**_**1hr.**_

**Figure 3:**
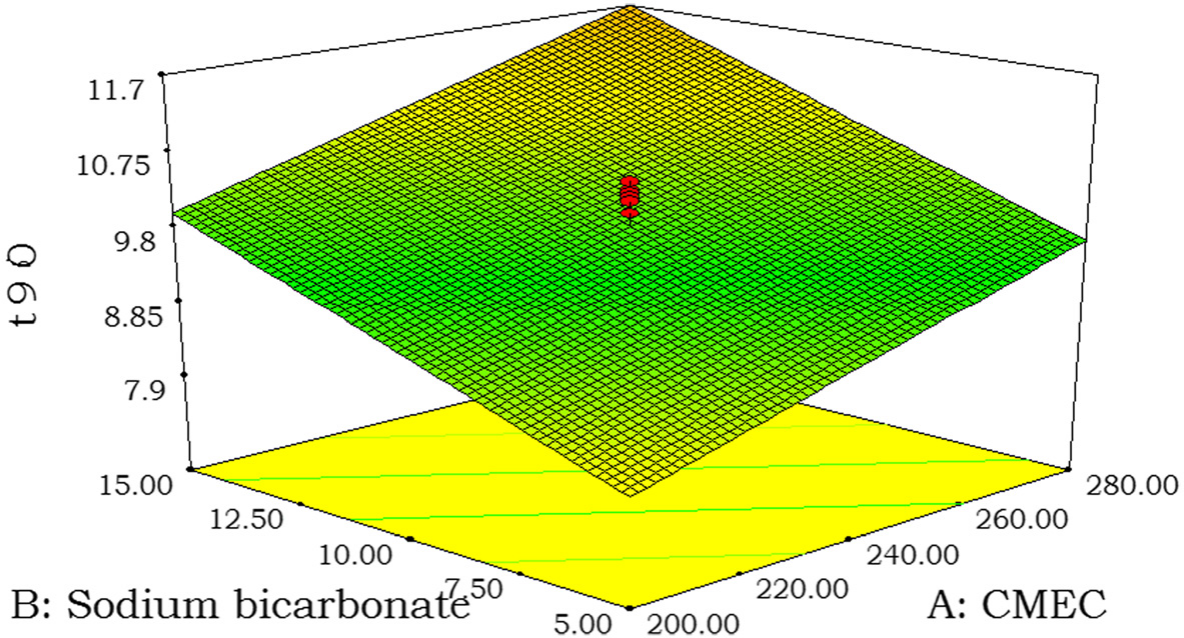
**Response surface plot for (A) the effect of CMEC and sodium bicarbonate concentrations on t**_**90.**_

**Figure 4:**
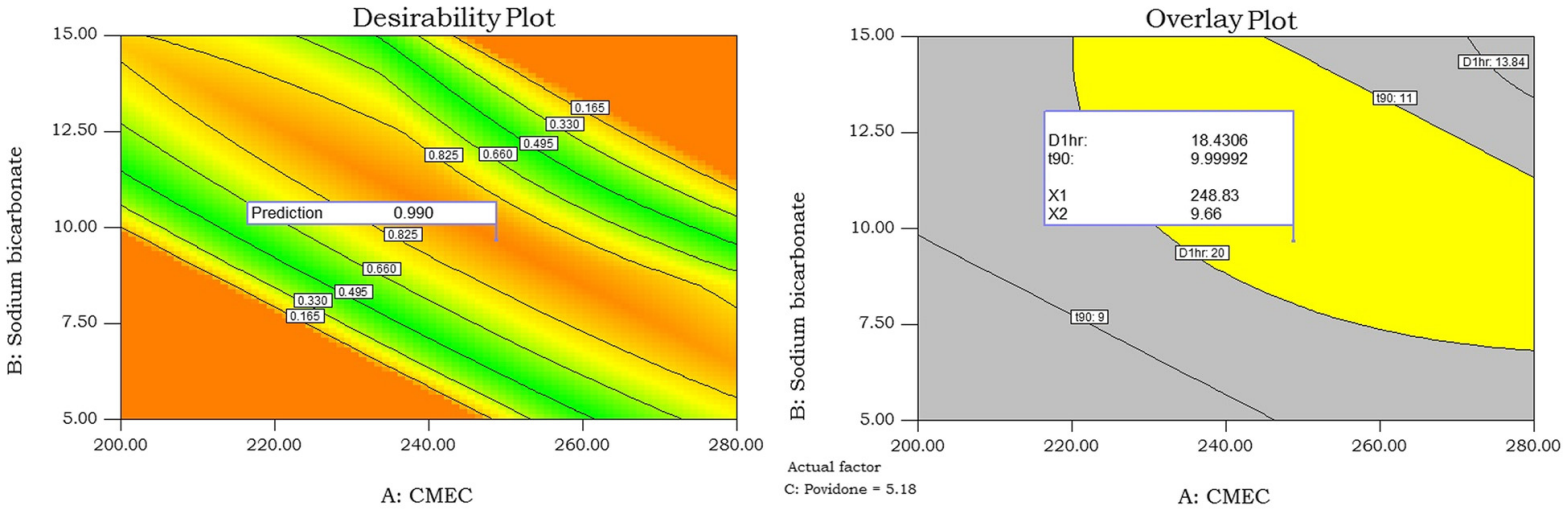
Desirability plot and Overlay plot for optimization of gastroretentive floating tablets of propranolol HCl.

The relationship between the dependent and independent variables was further elucidated by using response surface plots (Figure 2-3). These plots are useful in the study of the effects of factors on the response at one time and predict the responses of dependent variables at the intermediate levels of independent variables. Subsequently, a numerical optimization technique by the desirability approach (Figure 4) and graphical optimization technique by the overlay plot (Figure 4) were used to generate the new formulations with the desired responses.

### Validation of the experimental design

To validate the chosen experimental design, the resultant experimental values of the responses were quantitatively compared with those of predicted values and % relative error was calculated by the following equation;

(7) $$\%\;\text{Relative error}=\frac{\left(\text{Predicted value}-\text{Experimant value}\right)}{\text{Predicted value}}x\;100$$

### Drug interaction studies

#### Fourier transformation-infrared spectroscopy (FTIR)

FTIR is used to identify the drug excipient interaction. FTIR studies were performed on drug, polymer and statistically optimized formulation. Samples were analyzed by potassium bromide pellet method in an IR spectrophotometer (Shimadzu, FTIR 8700) in the region between 3500-500 cm^-1^.

#### Differential scanning calorimetry (DSC)

Differential Scanning Calorimetric analysis of drug, polymer and statistically optimized formulation were done using Differential Scanning Calorimeter (Mettler Toledo Star SW 8.10, Model no: DSC 822). In this process about 8-10 mg of the samples were weighed in aluminum pan and were heated under nitrogen atmosphere from 5 °C to 250 °C.

#### *In vivo* buoyancy studies

To confirm the spatial and temporary placement of floating drug delivery system, a variety of techniques have been used like string technique, endoscopy and gamma scintigraphy [17-20]. Of these techniques, X-ray technique was used to determine the gastric residence time of the tablets. In the present investigation X-ray studies were conducted for the evaluation of intragastric floating behavior of the statistically optimized GRFT of propranolol HCl both in fasted and fed states.

The *in vivo* X-ray evaluation of floating ability studies were carried out by administering GRFT of propranolol HCl containing barium sulfate (BaSO_4_) in humans in fasted and fed state.

Two healthy male subjects of mean age 25 ± 2 yrs (ranging from 23 to 27), mean weight 68 ± 10 Kg (ranging from 58 to 78 kg) and a mean height of 170 ± 5 cm (ranging from 165 to 175 cm) participated in this study. The volunteers were judged healthy on the basis of their previous medical history, physical examination and routine laboratory tests. Both subjects were presented with full details of the investigation, verbally and in written form, prior to providing written informed consent and the study was conducted under the guidance of radiologist. The study was approved from an independent Institutional Ethics Committee of Andhra University, Visakhapatnam (India).

The statistically optimized GRFT of propranolol HCl was administered to the two volunteers, one under fasted and another one under fed states.

1. Fasted state: The subject was fasted overnight and then swallowed the gastric floating tablet with 200 ml of water. No food was allowed up to 3 hrs of dosing. Subject was not allowed to lay down for sleeping. Every one hour a glass of water (200 ml) was given.

2. Fed state: The subject was fasted overnight and in the morning given a high calorie-high fat breakfast with a total calorie value of approximately 900 Cal. The floating tablet was administered with 200 ml of water after half an hour of the breakfast. The subject was not allowed to eat anything up to 6 hrs but given a glass of water (200 ml) every hour.

#### Preparation of GRFT for *in vivo* studies

Optimized GRFT of propranolol HCl containing barium sulfate (PCMECRsoB) for *in vivo* X-ray evaluation were prepared by direct compression method. The amount of propranolol HCl was reduced to 40 mg for incorporating the barium sulfate (40 mg) as radio opaque substance to maintain the constant weight of the tablet. Propranolol HCl (40 mg) was geometrically mixed with CMEC until a homogeneous blend was achieved. Barium sulfate (40 mg), Povidone and sodium bicarbonate were added to the above blend, mixed and lubricated with magnesium stearate (1%w/w). Final blend was directly compressed into tablets on a 16-station rotary tablet punching machine (M/s. Cad mach Machinery Co Pvt Ltd. India) using 9 mm round plain punches at hardness of 4-6 kg/cm^2^.

## Results and discussion

All the floating tablets were passed physicochemical tests like weight variation, assay and friability. Floating lag times of all the formulations were within the range of 280 to 720 sec (Table 2). As the concentration of sodium bicarbonate increases, the floating lag time found to be decreased.

The cumulative percent drug releases from GRFDDS prepared by central composite design with CMEC are shown in the Figure 1. From the results, it was observed that as the concentration of polymer increased along with concentration of sodium bicarbonate the drug release was retarded. This may be due to increased intensity of air pockets surrounding the jellified surface of the tablet. Increase in the concentration of the sodium bicarbonate at constant polymer concentration also retarded the drug release due to high intensity of the carbon dioxide gas pockets. Drug retardation was directly proportional to the concentration of Povidone which may be due to the formation of strong compactness between the particles [21].

All CMEC based formulations followed first order rate constant with non Fickian diffusion mechanism. (Table 3).

The responses of the floating tablets were fitted to linear, interaction and quadratic model using Design Expert software. As suggested by the software the responses floating lag time, and D_1hr_ is suggested to quadratic model and t_90_ is suggested to linear model (Table 4).

### Data analysis

By using semi synthetic polymer CMEC, 20 batches of formulations within the experimental design were prepared to obtain floating tablets which were evaluated for their floating lag time, D_1hr_ and t_90_. From the ANOVA data, the F value for the floating lag time was found to be 1.12 which indicates that the model is non-significant, whereas for other responses D_1hr_ and t_90_ the F value was found to be 16.04 and 21.28 respectively which indicates that both models are significant. The values of Prob > F less than 0.05 for all the responses except floating lag time are indicating that the models are significant. The response floating lag time exhibited Prob > F value 0.4269, which indicating model was not significant (Table 4). In the response observation for D_1hr_ A, B, C, B^2 and C^2, for t_90_ A, B and C was found to be significant model terms. For floating lag time no significant model terms were found (A: CMEC, B: Sodium bicarbonate, C: Povidone). The lack of fit F value for floating lag time, D_1hr_ and t_90_ was found to be 0.0111, 0.0003 and 0.0005 respectively implies that the lack of fit is significant. Similarly ‘R- squared’ value was also calculated for all responses. The ideal value is nearer to zero. ‘R- Squared’ value in the present model is near to zero which indicates towards a good model. In all the cases ‘Pred R squared’ values are in reasonable agreement with the ‘Adj R squared’ values except floating lag time (0.8769 & 0.5008 for D_1hr_ and 0.7621 & 0.6484 for t_90_). A negative ‘Pred R squared’ was observed for the floating lag time response which implies that the overall mean is a better predictor of this response than current model. In all the case ‘Adeq Precision’ values are in the range of 12 – 17 except floating lag time which indicates an adequate signal and the model can be used to navigate the design space. For floating lag time ‘Adeq Precision’ was found to be 3.49 which indicates an inadequate signal and we should not used this model to navigate the design space (Table 5). Even though lack of fit was significant for all the variables, the model was preceded further because of the positive results obtained with other parameters such as F value, values of Prob > F, ‘R- squared’, Pred R squared’ and ‘Adj R squared’ for D_1hr_ and t_90_ responses. Hence in the present investigation only D_1hr_ and t_90_ responses were taken as dependent variables and optimization was proceeding with these parameters.

The application of response surface methodology yielded the following regression equations which are an empirical relationship between the logarithmic values of floating lag time, D_1hr_ and t_90_. Test variables in coded units:

(8) $$\begin{array}{l} \begin{array}{l} Floating\;lag\;time=394.75-12.67*\text{A}+3.13*\text{B}-30.42*\text{C}-29.37*\text{A}*\text{B}+98.13*\text{A}*\text{C}+29.38*\text{B}*\text{C}\\ +63.02*{\text{A}}^{2}+29.61*{\text{B}}^{2}+1.32*{\text{C}}^{2} \end{array}\\ \begin{array}{l} D_{1hr}=+19.25-4.32*\text{A}-3.72*\text{B}-3.56*\text{C}-1.01*\text{A}*\text{B}+1.22*\text{A}*\text{C}-0.>48*\text{B}*\text{C}+1.47*{\text{A}}^{2}+1.78*{\text{B}}^{2}\;\\ +4.28*{\text{C}}^{2} \end{array}\\ t_{90}=9.83+0.85*\text{A}+1.02*\text{B}+0.63*\text{C} \end{array}$$

The contour and response surface plots for the all responses of all the formulation factors are shown in Figure 2-3. Contour and response plots of the response surface as a function of two factors at a time, holding all other factors at fixed levels, are more helpful in understanding both the main and the interaction effects of these two factors.

### Optimization

To optimize all the responses with different targets, a multi criteria decision approach like a numerical optimization technique by the desirability function and graphical optimization technique by the overlay plot were used (Figure 4). The optimized formulation was obtained by applying constraints on dependent variable responses and independent variables.

Optimized formulation was selected based on the criteria of less than 20% of the drug release at 1 hr (fixed by USP dissolution conditions [11]) and 90% of the drug released in between 10 to 11 hrs. Floating lag time was omitted in the optimization process as per the previous discussion. These constrains are common for all the formulations. The recommended concentrations of the independent variables were calculated by the Design Expert software from the above plots which has the highest desirability near to 1.0.

The optimum values of selected variables obtained by using Design Expert software was 248.88 mg of CMEC, 9.66% of sodium bicarbonate and 5.18% of Povidone for the development of GRFT of propranolol HCl.

### Evaluation and validation of optimized formulations

The optimized formulation fulfilled all the criteria of physicochemical properties. *In vitro *buoyancy and dissolution studies were carried out on the prepared optimized formulations for verification of the theoretical prediction. Observed responses and predicted values for D_1hr_ and t_90_ was found to be 17.21%, 10.3 hrs and 18.43%, 10 hrs respectively. The % relative error between the predicted values and experimental values of each response was calculated and the values were found to be 2.55% and 3.0% for D_1hr_ and t_90_ respectively. From the results, it was concluded that these experimental findings are in close agreement with the model predictions which confirmed the predictability and validity of the model.

### Drug interaction studies

#### Fourier transformation-infrared spectroscopy (FTIR)

**Figure 5:**
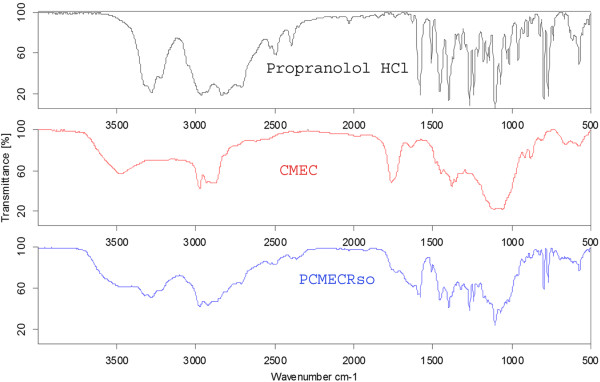
FTIR spectra of propranolol HCl, CMEC and PCMECRso.

The FTIR spectrum of propranolol HCl, CMEC and Optimized formulation are shown in Figure 5. Propranolol HCl showed characteristic secondary amine –N–H stretch at 3280 cm^-1^, C-H stretch at 2964 cm^-1^, Aryl C = C stretch at 1579 cm^-1^, Aryl 0-CH_2_ asymmetric stretch at 1240 cm^-1^, Aryl 0-CH_2_ symmetric stretch at 1030 cm^-1^ and the peak at 798 cm^-1^ due to alpha- substituted naphthalene [22].

The FTIR spectrum of CMEC showed the characteristic alcoholic –OH stretch at 3476 cm^-1^, C-H stretch at 2976 cm^-1^, -C = O stretch at 1761 cm^-1^ and -C-O-C asymmetric stretch at 1378 cm^-1^.

Statistically optimized CMEC based formulation (PCMECRso) showed all the characteristic peaks of propranolol HCl with minor shifts in its FTIR spectrum. This spectrum showed secondary amine –N–H stretch at 3280 cm^-1^, C-H stretch at 2974 cm^-1^, Aryl C = C stretch at 1579 cm^-1^, Aryl 0-CH_2_ asymmetric stretch at 1241 cm^-1^, Aryl 0-CH_2_ symmetric stretch at 1031 cm^-1^ and the peak at 797 cm^-1^ due to alpha- substituted naphthalene.

#### Differential scanning calorimetry

**Figure 6:**
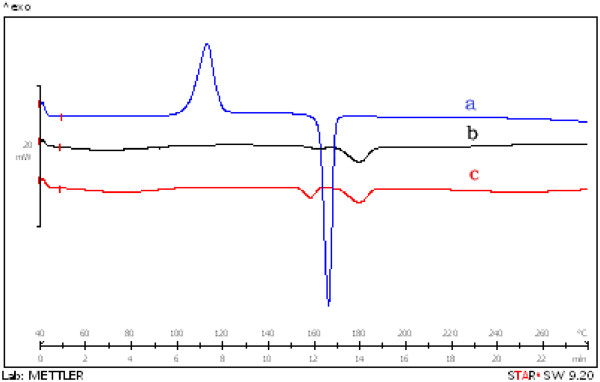
DSC thermogram of a) propranolol HCl, b) CMEC and c) PCMECRso.

DSC thermogram of propranolol HCl, CMEC and PCMECRso are shown in the Figure 6. The DSC thermogram of CMEC showed a sharp endothermic peak at 183.5 °C that corresponds its melting point. From the results, it was observed that the thermogram of statistical optimized formulation PCMECRso showed sharp endothermic peaks at 163.2 °C and 183.1 °C represents drug and polymer respectively, which indicated that slight decrease in the energy change of melting endotherm, which confirms minor extent of reduction in the crystallinity of the drug but not a significant reduction.

The absence of any changes in the FTIR spectra and DSC thermogram for the selected formulation indicated no chemical interaction between the CMEC and drug.

#### *In vivo* buoyancy studies

This study was aimed to examine whether the floating tablet system could buoyant and retain in the stomach. A radiological method was adopted to monitor the developed gastro retentive floating tablets in the gastric region of humans in different feeding conditions. The GRFT remained buoyant on gastric content under both fasted and fed states in volunteers participated in the present study. However, a difference in floating and gastric retention time was obeyed according to the feeding conditions given in Table 6.

**Table 6:** ***In vivo*** **residence time of the optimized GRFT of propranolol HCl containing barium sulfate (PCMECRsoB)**

**Time (hrs)**	**Position of the tablet in GIT**	
	**Fed state**	**Fasted state**
0.5	Stomach	Stomach
2	Stomach	Stomach
4	Stomach	Small intestine
6	Stomach	Disappeared from gastric region
8	Disappeared from gastric region	

**Figure 7:**
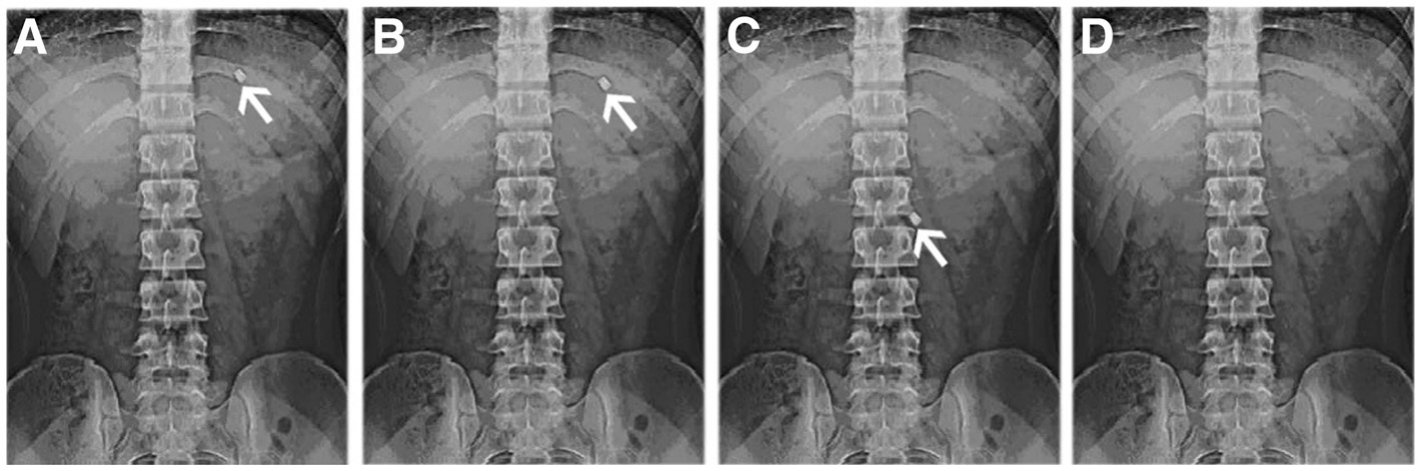
X- ray photographs of gastric floating tablets of PCMECRsoB containing propranolol HCl under fasted state after (a) 0.5 hrs (b) 2 hrs (c) 4 hrs (d) 6 hrs.

In the fasted state, the floating tablets were observed to be buoyant on the gastric fluid up to at 2 hr as shown in Figure 7 (a&b) and were observed in the small intestine after 4 hrs as shown in Figure 7 (c) and was disappeared at 6th hr as shown in Figure 7 (d). Therefore, in such condition, the floating property did not enhance gastric retention time (GRT). The rapid emptying was attributed to periods of strong contractile activity, which occur under fasting conditions every 1.5 to 2 hrs, and effectively sweep undigested material from the stomach [20,23]. As a result of this activity, dosage form administered to fasted subjects could be emptied as rapidly as within an hour or two, depending on the presence of the strong motor induced contractile activity.

**Figure 8:**
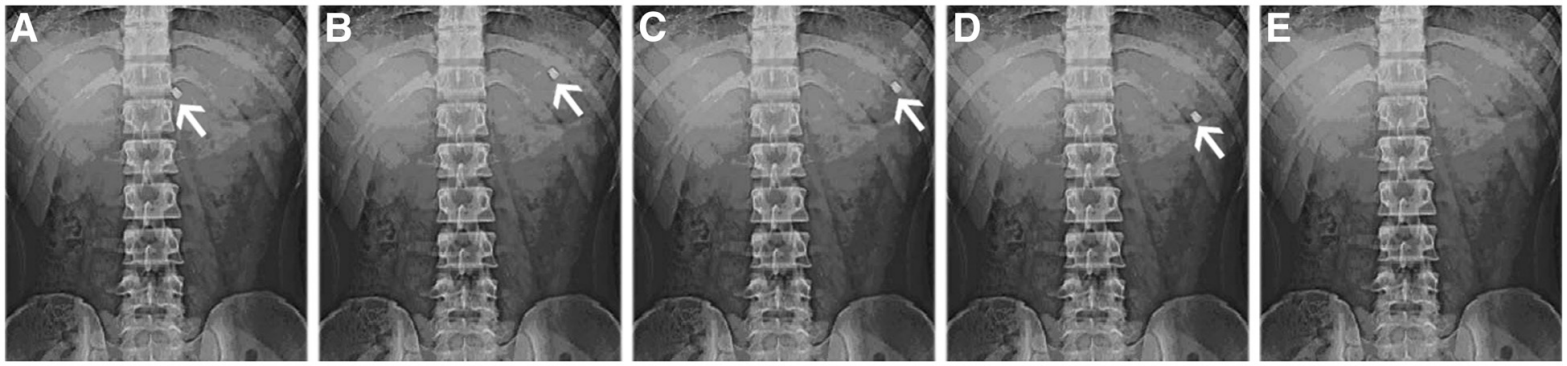
X- ray photographs of gastric floating tablets of PCMECRsoB containing propranolol HCl under fed state after (a) 0.5 hrs (b) 2 hrs (c) 4 hrs (d) 6 hrs (e) 8 hrs.

In the fed state after the high calorie high fat breakfast, the GRFT was observed to be buoyant on the gastric contents up to 6 hrs after administration as shown in Figure 8 (a) at 0.5 hrs, 8 (b) at 2 hrs, 8 (c) at 4 hrs, 8 (d) at 6 hrs and disappeared at 8^th^ hr shown in Figure 8 (e).

Therefore, in the fed condition, the floating system showed a GRT prolonged by about 5 to 6 hrs over the fasted state.

The evaluation of the GRFT of propranolol HCl intragastric behavior in humans, showed the actual floatability of the tablet on the gastric content.

This study has demonstrated that in the fasted state under the influence of strong motor activity (the migrating myoelectric complex), there was no enhancement of GRT of gastro retentive floating tablet, where as there was a prolonged GRT of approximately 6 hrs in a fed state.

## Conclusion

Thus the present study clearly indicated the applicability of the statistical optimization techniques for the prediction of the optimized concentrations of the excipients that influence the product parameters. These theoretical predictions can be verified for their experimental success as in the present case. The statistical optimization reduces the number of experiments to be carried for obtaining formulation with desired properties. Moreover the optimization is also useful in reducing the concentrations of the excipients to their optimum levels avoiding unnecessary wastage of excipients and thereby reducing the cost of the final product. The intragastric behavior of statistically optimized GRFT of propranolol HCl in humans, showed the floatability of the tablet on the gastric content. *In vivo* evaluation demonstrated no enhancement of GRT of gastro retentive floating tablet in fasted state, where as there was a prolonged GRT of approximately 6 hrs in the fed state. From the results, it is concluded that CMEC is novel semi synthetic polymer suitable for the development of GRFT of propranolol HCl.

## Competing interests

The author(s) declare that they have no competing interests.

## Authors’ contributions

MVS: The corresponding author involved in the literature survey, procurement of excipients, plan of research, statistical design, carrying out the bench work, statistical interpretation and drafting of the final manuscript. NSR: Co- research scholar who was involved in conducting dissolution studies and physicochemical characterization of the formulations. SAS: Co-research scholar involved in the analytical method development and interpretation of the FTIR & DSC studies. BJR: Senior research scholar involved in the *in vivo* buoyancy characterization of the formulations and he gave valuable suggestions for drafting the manuscript. KVRM: Research guide, who gave valuable suggestions in the design of experimental formulas, interpretation of the statistical data, critical review of the manuscript for intellectual content, vital and crucial review and approval of the final manuscript to be published. He also granted me permission to carry out research activities along with use of the equipment in the laboratory. All the above authors read and approved the final manuscript.
